# Complete Genome Sequence of the Halophilic Methylotrophic Methanogen Archaeon *Methanohalophilus portucalensis* Strain FDF-1^T^

**DOI:** 10.1128/genomeA.01482-17

**Published:** 2018-01-18

**Authors:** Stéphane L’Haridon, Erwan Corre, Yue Guan, Manikandan Vinu, Violetta La Cono, Michail Yakimov, Ulrich Stingl, Laurent Toffin, Mohamed Jebbar

**Affiliations:** a Université de Brest (UBO), Institut Universitaire Européen de la Mer (IUEM), UMR6197, Laboratoire de Microbiologie des Environnements Extrêmes (LM2E), Plouzané, France; b CNRS, IUEM-UMR 6197, Laboratoire de Microbiologie des Environnements Extrêmes (LM2E), Plouzané, France; c Ifremer, UMR6197, Laboratoire de Microbiologie des Environnements Extrêmes (LM2E), Plouzané, France; d CNRS, Université Pierre et Marie Curie, Station Biologique de Roscoff, Plateforme ABiMS, Roscoff, France; e Red Sea Research Center, King Abdullah University of Science and Technology, Thuwal, Saudi Arabia; f Institute for Coastal Marine Environment CNR, Messina, Italy; g Department for Microbiology and Cell Sciences, University of Florida, UF/IFAS Fort Lauderdale Research and Education Center, Davie, Florida, USA

## Abstract

We report here the complete genome sequence (2.08 Mb) of *Methanohalophilus portucalensis* strain FDF-1^T^, a halophilic methylotrophic methanogen isolated from the sediment of a saltern in Figeria da Foz, Portugal. The average nucleotide identity and DNA-DNA hybridization analyses show that *Methanohalophilus mahii*, *M. halophilus*, and *M. portucalensis* are three different species within the *Methanosarcinaceae* family.

## GENOME ANNOUNCEMENT

*Methanohalophilus portucalensis* strain FDF-1^T^ (DSM 7471^T^, OCM 59) was described as a new species by Boone et al. (1). The strictly anaerobic species is able to produce methane by reducing methyl compounds and grows optimally at 40°C within a pH range of 6.5 to 7.5 and a salinity range of 0.5 to 2 M NaCl.

The complete genome of *M. portucalensis* was sequenced using a combination of the following three sequencing approaches: a 300-bp paired-end library sequenced on an Illumina MiSeq platform (Bioscience Core Lab, KAUST, Thuwal, Saudi Arabia), a 100-bp paired-end library sequenced on an Illumina HiSeq platform (Beckman Coulter Genomics, Inc., Danvers, MA, USA), and a PacBio RS library (Genotoul, Toulouse, France). The 13,835,892 paired reads of 300 bp were quality trimmed (Q30) and *de novo* assembled into contigs using SPAdes version 3.6.1 (2). The 17 resulting contigs were then scaffolded with SSPACE version 3.0 (3) using the 60,686,211 paired-end reads of 100 bp, leading to an intermediate version of the assemblage containing 9 scaffolds. A final scaffolding step was performed with SSPACE LongRead version 1.1 (4) using the 317,258 PacBio RS-filtered subreads. We finally obtained 3 scaffolds with a total size of 2,084,275 bp (without an unspecified base) and an average coverage of approximately 5,300×. Finally, a fully circularized version of the genome with expected gaps between oriented scaffolds was produced after comparison with the *M. mahii* genome using CONTIGuator (5).

The *M. portucalensis* genome consists of a circular chromosome of 2,084,875 bp with a GC content of 41.95%. A total of 2,198 coding DNA sequences were identified with the MaGe platform (6, 7), as well as 2 6S-23S operons, 3 5S rRNAs, 46 tRNAs, and 4 miscellaneous RNAs.

The *M. portucalensis* genome size is close to that of *M. mahii* strain SLP^T^ (2,012,424 bp) (8) and *M. halophilus* strain Z-7982 (2,022,959 bp) (9). *M. mahii*, *M. halophilus*, and *M. portucalensis* are physiologically very similar, and their separation into three different species was previously based on DNA reassociation and electrophoretic analysis of whole-cell proteins and on the need to maintain the genus *Methanohalophilus* for taxonomic stability (1). The average nucleotide identity (ANI) scores are 92.59% (SD, 2.91) between *M. halophilus* and *M. portucalensis*, 92.59% (SD, 2.91) between *M. halophilus* and *M. mahii*, and 92.59% (SD, 2.91) between *M. mahii* and *M. portucalensis*. An ANI score below 95% has been defined for the delineation of a new species (10). Thus, the ANI score comparisons indicate that the three strains are on the boundary of the species delineation. Afterward, we calculated the *in silico* DNA-DNA hybridization (DDH) values using the genome-to-genome distance calculator GGDC2.1 (11), which indicated values of 44.8% between *M. halophilus* and *M. mahii*, 44.4% between *M. portucalensis* and *M. mahii*, and 50.50% between *M. halophilus* and *M. mahii*. The ANI and DDH values confirm that *M. mahii*, *M. halophilus*, and *M. portucalensis* represent three phylogenetically closely related species.

### Accession number(s).

This whole-genome sequence has been deposited at GenBank under the accession number CP017881.
